# Evaluation of an outbred mouse model for *Francisella tularensis* vaccine development and testing

**DOI:** 10.1371/journal.pone.0207587

**Published:** 2018-12-11

**Authors:** Raju Sunagar, Sudeep Kumar, Prachi Namjoshi, Sarah J. Rosa, Karsten R. O. Hazlett, Edmund J. Gosselin

**Affiliations:** Department of Immunology & Microbial Disease, Albany Medical College, Albany, NY, United States of America; Midwestern University, UNITED STATES

## Abstract

*Francisella tularensis* (*Ft*) is a biothreat agent for which there is no FDA-approved human vaccine. Currently, there are substantial efforts underway to develop both vaccines and the tools to assess these vaccines. Tularemia laboratory research has historically relied primarily upon a small number of inbred mouse strains, but the utility of such findings to outbred animals may be limited. Specifically, C57BL/6 mice are more susceptible than BALB/c mice to *Ft* infection and less easily protected against challenge with highly virulent type A *Ft*. Thus, depending on the inbred mouse strain used, one could be misled as to which immunogen(s)/vaccine will ultimately be effective in an outbred human population. Accordingly, we evaluated an outbred Swiss Webster (SW) mouse model in direct comparison to a well-established, inbred C57BL/6 mouse model. Mucosal vaccination with the live, attenuated *Ft* LVS superoxide dismutase (*sodB*) mutant demonstrated significantly higher protection in outbred SW mice compared to inbred C57BL/6 mice against *Ft* SchuS4 respiratory challenge. The protection observed in vaccinated outbred mice correlated with lower bacterial density, reduced tissue inflammation, and reduced levels of pro-inflammatory cytokine production. This protection was CD4^+^ and CD8^+^ T cell-dependent and characterized by lower titers of serum antibody (Ab) that qualitatively differed from vaccinated inbred mice. Enhanced protection of vaccinated outbred mice correlated with early and robust production of IFN-γ and IL-17A. Neutralizing Ab administered at the time of challenge revealed that IFN-γ was central to this protection, while IL-17A neutralization did not alter bacterial burden or survival. The present study demonstrates the utility of the outbred mouse as an alternative vaccination model for testing tularemia vaccines. Given the limited MHC repertoire in inbred mice, this outbred model is more analogous to the human in terms of immunological diversity.

## Introduction

*Francisella tularensis* (*Ft*) is a gram negative intracellular pathogen and Tier 1 select agent [1–3]. However, despite extensive research and investment over the last 15 years, there remains no FDA-approved vaccine.

The murine model has been instrumental in uncovering immunological mechanisms that underlie protective or non-protective immune responses against various infectious pathogens including *Ft*. Inbred mouse strains are of interest as the majority of the vaccination and infection studies are done using models such as C57BL/6 or BALB/c mice. Importantly however, the genetic background of the mice can have a significant impact on the response to vaccination and challenge [4–7]. It has been demonstrated that C57BL/6 mice are less easily protected (compared to BALB/c mice) against virulent type A *Ft* challenge; this has been attributed to waning T cell immunity in C57BL/6 mice [8]. It has also been demonstrated that C57BL/6 mice favor the development of a Th2 phenotype rather than the more protective Th1 response in the lungs [4, 9]. Together, these findings suggest vaccinated C57BL/6 mice fail to develop a sufficiently protective immune response to subsequent *Ft* infection as compared to BALB/c mice.

The differential susceptibility between inbred mouse strains is likely due in part to the very limited and differing MHC repertoire present in each specific mouse strain [10, 11] all of which could lead to erroneous conclusions regarding vaccine efficacy in outbred populations such as humans [12]. Successful vaccine development to *Ft* will also require analysis of vaccine efficacy in more genetically diverse animal models [13]. Specifically, the more heterogeneous immune responses in an outbred animal model are more likely to successfully predict vaccine efficacy for outbred humans. The latter is supported by the observations that an outbred mouse model of pneumonic plague, influenza and bacterial co-infection for example shows similar disease pathology to that observed in humans [14, 15], as well as the observation that pre-clinical modeling of the immune response seems most analogous to humans in the outbred animals[16–18]. Regarding *Ft* however, there is a paucity of information on *Ft* vaccine development in outbred mouse models [19–22].

We initiated *Ft* vaccine studies using the outbred [Swiss Webster (SW)] model, which in a recent study of pneumonic plague pathology, showed similar pathology to that observed in humans [14]. When comparing immunity and protection against challenge in SW versus C57BL/6 mice, SW mice were more resistant to virulent *Ft* SchuS4 challenge and responded more favorably to vaccination. Furthermore, improved immunity generated in SW mice correlated with lower inflammation and tissue damage and appeared dependent on CD4 and CD8 T cell responses.

## Materials and methods

### Mice

**Fig 1:**
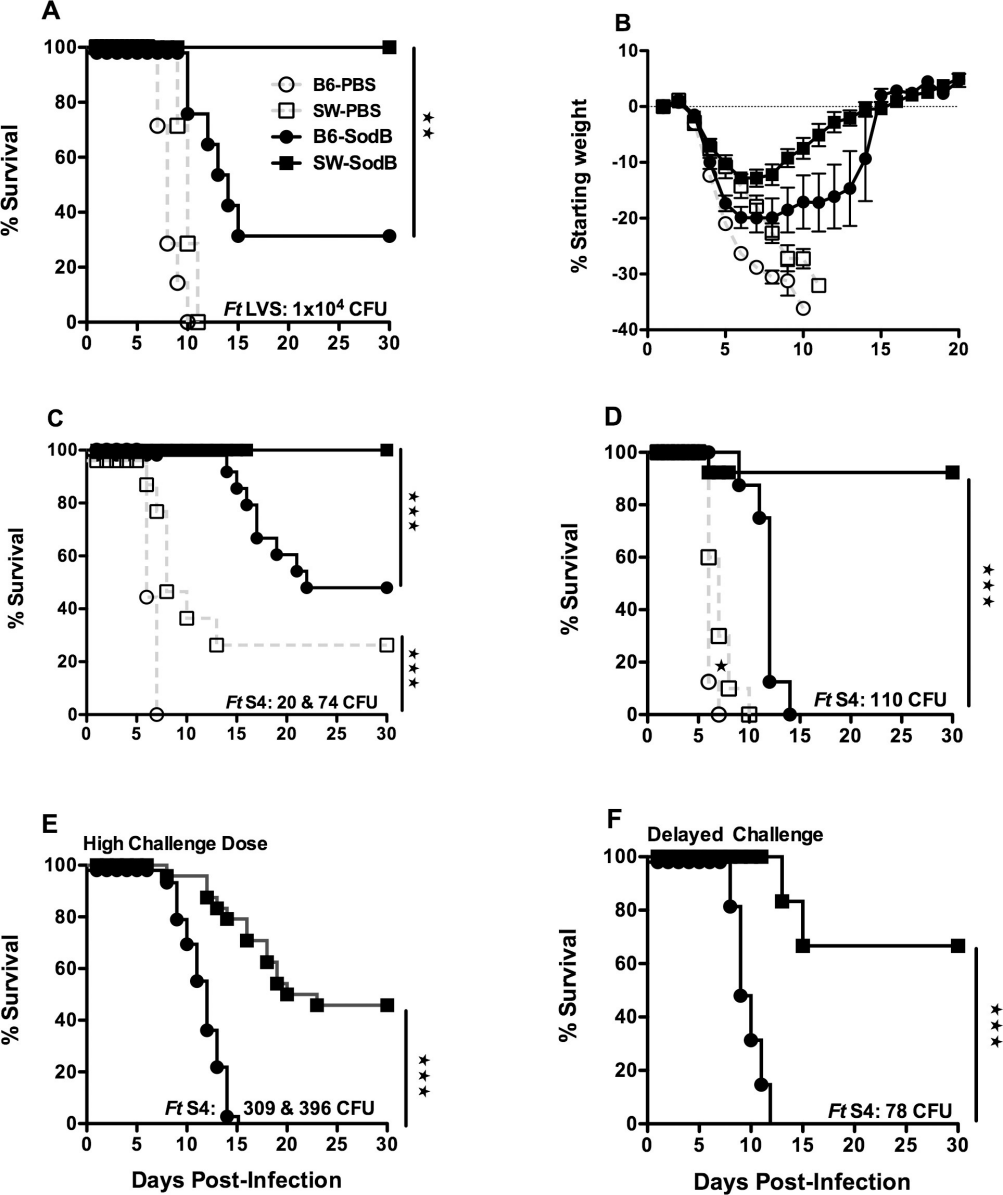
Outbred SW mice exhibit superior protection against pulmonary tularemia induced mortality versus inbred C57BL/6 mice. SW and C57BL/6 male and female mice were primed and boosted i.n. on days 0 and 21 with 1500 ng of i*Ft-sodB* or PBS prior to challenge i.n. on day 35 with 5 x LD_50_ of *Ft* LVS. Mice were monitored for survival (**A**) and weight-loss (**B**) for 30 d. For panels **C-F**, SW and C57BL/6male and female mice were vaccinated once i.n. with 1x10^3^ CFU of attenuated *Ft* LVS *sodB* mutant (closed symbols) or PBS (open symbols). Mice were challenged i.n. with *Ft* SchuS4 on day 21(**C**, **D**, and **E**) or day 90 (**F**). Panels A, B, D and F represent one experiment containing 8 mice/group, while panels C & E are combined results from two independent experiments with a total of 16 mice/group. Actual challenge doses (CFU) are indicated for each experiment. **p < 0.01 and ***p < 0.001.

Specific pathogen-free 6-to-8-week-old male and female C57BL/6 mice were from Taconic Farms (Hudson, NY). Male and female Swiss Webster Crl:CFW (SW) mice were from Charles River Laboratories, Inc. (Portage, MI). Survival studies included male and female mice (Fig 1A–1F), while remaining experiments were performed using female mice. Mice were housed in sterile microisolator cages in the animal biosafety level 2 (ABSL-2) and ABSL-3 facilities at the Albany Medical Center. The use of animals and protocols were approved by the Institutional Animal Care and Use Committee (IACUC) of Albany Medical College (Protocol Number 17–09003 and 18–04003). Death is used as an endpoint, because euthanizing mice in a pre-morbid state could result in an incorrect interpretation of the studies, specifically as it applies to the efficacy of a particular vaccine. In short, it is possible pre-morbid mice may recover as a result of vaccination, if not sacrificed prematurely. In all experimental procedures, efforts were made to minimize pain and suffering.

### Bacteria

Attenuated *Ft* LVS *sodB*, [superoxide dismutase B mutant [23]] was grown on BHI plates for 48 hr, *Ft* SchuS4 *clpB* [ClpB mutant [24]], *Ft* LVS, *Ft* SchuS4,were cultured aerobically at 37°C on modified Mueller-Hinton (MH) or Brain-Heart Infusion (BHI) broth (Becton Dickinson, Sparks, Md) used for immunization and infection studies. Inactivated *Ft* (i*Ft*) was generated as described [25].

### Vaccination and challenge studies

Mice were anesthetized as previously described [26]. For i*Ft* LVS *sodB* vaccination, mice were immunized on day 0 and boosted on day 21 intranasally (i.n.) with either 20 μl of PBS or 1500 ng of i*Ft* in 20 μl of PBS. On day 35 the animals were challenged i.n. with 1x10^4^ CFU (5 x LD_50_) of *Ft* LVS. For live *Ft* vaccination, BHI grown *Ft* LVS *sodB* mutant (1 x 10^3^ CFU) were administered i.n. in 20 μl of PBS on day 0 and mice were challenged i.n. with MHB-grown *Ft* SchuS4 (20–400 CFU) on day 21. In all cases, mice were monitored for survival 30 days post-challenge.

### Bacterial burden

Following immunization and challenge, mice were euthanized at various times by i.p. injection of pentobarbital (Fort Dodge laboratories, IA). Lungs, and spleens were homogenized in PBS using a MiniBeadBeater-8 (BioSpec Products, Bartlesville, OK). Tissue homogenates and bronchoalveolar lavage fluid (BALF) were diluted 10-fold in sterile PBS and plated onto chocolate agar plates and incubated at 37°C for 2–3 days.

### Histopathology

Whole lung tissue was excised and fixed in 10% neutral-buffered formalin. Fixed tissues were processed by standard histological procedures and 4μm-thick sections were cut and stained with hematoxylin and eosin (HE). Disease severity was assessed based upon cellular infiltration, and necrosis of alveolar septa,

### Serum LDH and BALF total protein

Serum concentrations of lactate dehydrogenase (LDH) were measured as described [26]. Total protein in BALF was measured via bicinchoninic acid (BCA) assay (Sigma-Aldrich, St. Louis, Missouri). Briefly, 25 μl of each standard or unknown sample were mixed with 200 μl of BCA reagent and incubated at 37°C for 30 minutes and read at 562 nm. Fold changes were calculated based on mean BALF protein or serum LDH concentrations from un-infected C57BL/6 or SW mice.

### Cytokine quantification

Tissue homogenates were obtained as indicated above when measuring bacterial burden. Supernatants were then collected and stored at -20°C for cytokine analysis. Luminex assay was performed to determine *in vivo* cytokine levels of interferon-gamma (IFN-γ), interleukin-6 (IL-6), tumor necrosis factor-α (TNF-α), interleukin-17 (IL-17), interleukin-22 (IL-22), interleukin-10 (IL-10), interleukin-12p40 (IL-12p40), and monocyte chemoattractant protein-1 (MCP-1) to assess inflammation.

### *Ft*-specific Ab levels

Anti-*Ft* Ab production in response to immunization and/or *Ft* infection in immunized mice was measured through enzyme linked immunosorbent assay (ELISA). Briefly, ELISA plates were coated overnight at 4°C with 50 μl/well of inactivated *Ft* SchuS4 [5x10^7^ CFU/ml in carbonate buffer [4.3g/l sodium bicarbonate and 5.3 g/l sodium carbonate (Sigma-Aldrich, St. Louis, Missouri) at pH 9.4]. Plates were then blocked at 4°C for 2 hours with 200 μl/well of PBS containing 5% BSA and 0.02% sodium azide. Subsequently three-fold dilutions of sera (starting with 1:50) were added to the plates (50 μl/well) and incubated for 2 hours at 4°C. After washing, alkaline phosphatase conjugated anti-mouse Ab specific for IgG, IgA (Sigma-Aldrich, St. Louis, Missouri), or IgG2c (Abcam, Cambridge, MA), were added and incubated for 1 hours at 4°C. Following secondary Ab incubation and washing alkaline phosphatase substrate (Sigma-Aldrich, St. Louis, Missouri) of 100 μl/well was added and plates were read at 405 nm using microplate reader following a 5-second (sec) shake.

### SDS-PAGE and western blot analysis

For western blot analysis, *Ft* LVS and *Ft* SchuS4 ΔClpB [24] were grown in BHI-broth. Whole cell samples containing 10 μg of *Ft* protein (~1 x 10^8^ cells) were mixed with Laemmli sample buffer and boiled for 10 minutes prior to resolution through 4–12% gradient SDS-PAGE. Resolved samples were transferred to nitrocellulose membranes. Membrane sections were blocked and incubated with mouse sera overnight. Biotinylated goat anti-mouse IgG (H+L) secondary Ab followed by Streptavidin-conjugated HRP (Southern Biotech, Birmingham, AL) was used for detection. Development of the chemiluminescent substrate (Super Signal West Pico, Pierce, Rockford, IL) was visualized using a BioRad ChemiDoc Touch Imaging System.

### *In vivo* T cell depletion and cytokine neutralization

Vaccinated mice were treated i.p. with 500 μg of anti-CD4 (clone GK1.5; Bio X Cell, West Lebanon, NH) or anti-CD8 (clone 53–6.7; Bio X Cell), or rat IgG2b (clone LTF-2) on days -4, -1, 2, 5, and 8 relative to *Ft* SchuS4 challenge [27]. Alternatively, mice were treated with anti-IFN-γ (clone XMG1.2) or anti-IL-17A (clone 17F3) on day’s -1, 1, 3, and 5 relative to *Ft* SchuS4 infection. Control groups received non-specific rat IgG2b (clone LTF-2) or mouse IgG1 (clone MOPC-21; Bio X Cell).

### Statistical analysis

For each experiment, mouse group size was determined by a power analysis necessary to achieve at least an 80% power to detect a difference in immune responses when comparing 2 groups, if the true difference in means between the groups is at least 1.5. Power calculations are based on standard deviations obtained from the PI’s previous studies using similar methods/animal models. Student’s t-test or two-way ANOVA with Mann-Whitney two-tailed test was used for statistical comparisons between groups. In the case of survival analysis, the Log-rank (Montel-Cox) test was used with a *P* value of < 0.05 considered significant. Statistical analysis and data compilation were using GraphPad prism (v6.0).

## Results

### Differential protection of outbred SW versus inbred C57BL/6 mice following vaccination

Initially, we compared protection induced by i*Ft* or live *Ft* LVS *sodB* immunogens, which have been shown to induce partial protection against *Ft* LVS [26] and virulent *Ft* SchuS4 challenge respectively [23]. All PBS-immunized C57BL/6 and SW mice succumb to *Ft* LVS infection with minor (2 d) differences in the mean time to death (MTD). However, a marked difference was observed among i*Ft*-vaccinated mice. Specifically, we observed 100% protection for vaccinated SW mice whereas only 33% of the vaccinated C57BL/6 mice survived and these displayed increased weight loss (Fig 1A and 1B). We extended these studies to include challenge with virulent *Ft* SchuS4. Following vaccination with live *Ft* LVS *sodB*, in SW mice we observed 100% protection against 20 & 76 CFU *Ft* SchuS4, as opposed to 50% protection for inbred mice. Interestingly, unvaccinated SW mice challenged with low doses of *Ft* SchuS4 also exhibited increased (30%) survival versus C57BL/6 mice (Fig 1C). Further following 30 days post-challenge, *Ft* was detected in low numbers in the surviving naïve SW mice, confirming these mice were infected with *Ft*. Further, when we increased the challenge dose to 110 CFU, all PBS-immunized (outbred and inbred) along with *sodB*-immunized inbred mice succumbed to SchuS4 challenge while 94% of the vaccinated SW mice survived (Fig 1D). When the challenge dose was further increased to 309 & 396 CFU, all vaccinated inbred mice succumb to infection by day 15 (MTD of 12 days) while a 45%of the outbred mice survived (Fig 1E). To examine the durability of the protective response, we next delayed challenge until 9 weeks post-immunization. All vaccinated C57BL/6 mice succumb to infection by day 12 (MTD of 9.5 days), while 62% of the vaccinated outbred mice survived a 78 CFU *Ft* SchuS4 challenge (Fig 1F). Given our past finding that gender impacts protection following vaccination in C57BL/6 mice, we compared male and female outbred mice survival. However, in contrast to C57BL/6 inbred mice, the vaccinated SW outbred mice did not exhibit the gender differences in protection against *Ft* SchuS4 challenge (S1 Fig). Importantly, although some studies have examined how the gender differences in protection may be a reflection of limited MHC repertoire, such studies are very limited and the details of this relationship remain unclear at this time. Never the less, studies have found a relationship between HLA repertoire and with disease outcome [28]. In addition, a very recent study has demonstrated sex bias in MHC I-associated shaping of the adaptive immune system in that the biological sex of an individual can influence the HLA-mediated T cell selection and expansion [29], which can ultimately influence T cell response and disease outcome. However, in contrast to C57BL/6 inbred mice, the vaccinated SW outbred mice did not exhibit the gender differences in protection against *Ft* SchuS4 challenge, which suggests the differences we previously observed in inbred C57BL/6 mice may be a reflection of their more limited MHC repertoire.

### Vaccinated outbred SW mice are better protected against bacterial replication and tissue damage following *Ft* SchuS4 challenge

**Fig 2:**
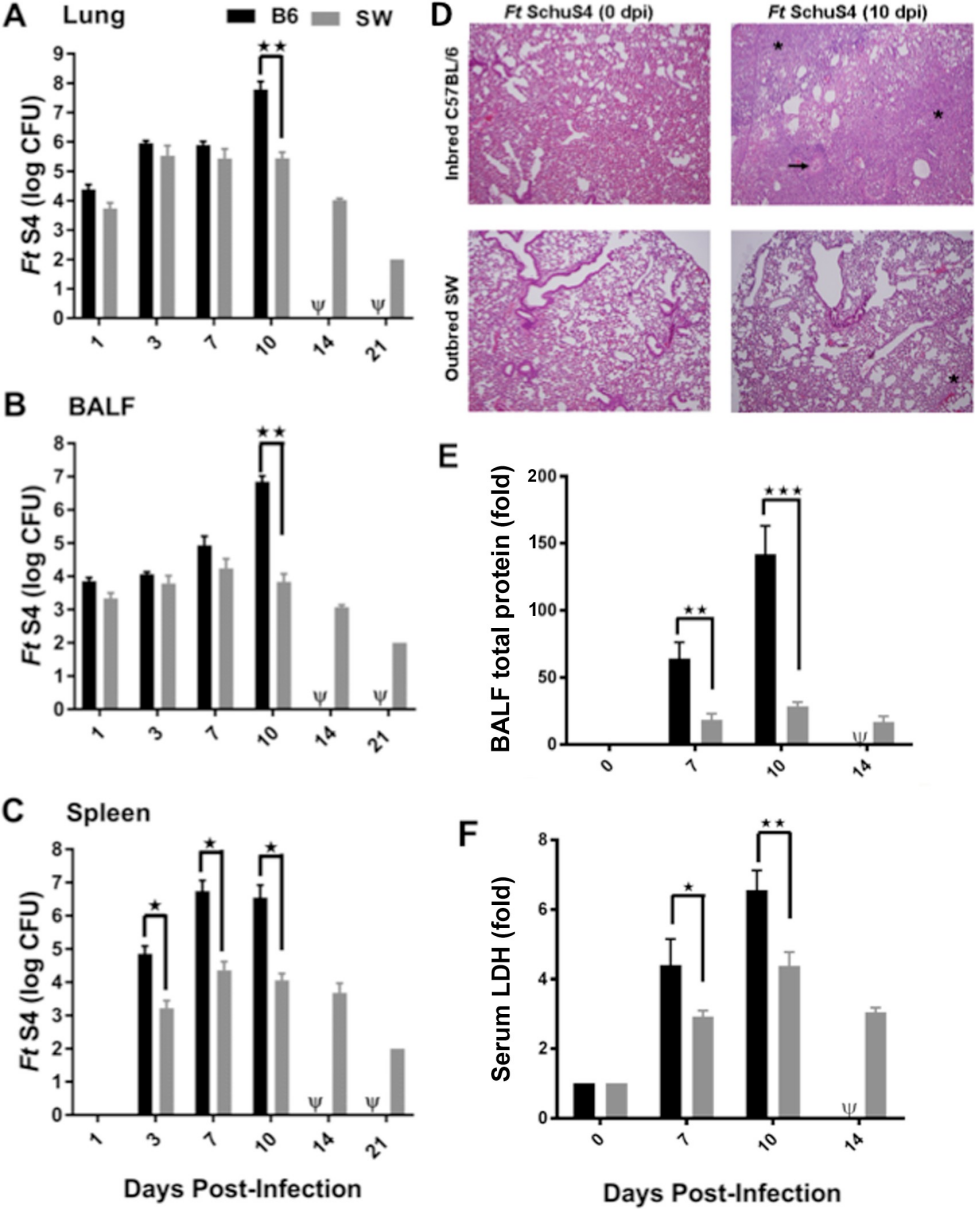
Outbred SW mice are better protected against bacterial replication and tissue damage following *Ft* SchuS4 challenge, as compared to that of inbred C57BL/6 mice. SW and C57BL/6 female mice were immunized i.n. with 1x10^3^ CFU of attenuated *Ft* LVS *sodB* mutant. Mice were then challenged on day 21 i.n. with 94 CFU of *Ft* SchuS4. Lungs (**A**), BALF (**B**), and spleen (**C**) were analyzed for bacterial burdens on 1, 3, 7, 10, 14, and 21 days post-challenge. Each point represents the mean +/- SE of 3 mice sacrificed per time point, these data are representative of two independent experiment of total of six mice, *p < 0.05, **p < 0.01. Histology of lungs (HE, 400X) from naive and *sodB*-vaccinated mice prior to *Ft* SchuS4 challenge (day 0) and at day 10 post-infection are shown in panel (**D**). Inflammatory foci in the lungs are denoted by a star (*) and necrosis by an (→). BALF total protein levels (**E**) and serum LDH (**F**) were quantified on indicated days post-challenge. Each bar represents mean ± SE (error bar) of two independent experiments with a total of six mice per group, *p < 0.05, **p < 0.01 and ***p < 0.001. Data shown are representative of two independent experiments. All vaccinated inbred mice died, as indicated by Ψ on the *x*-axis.

These differences prompted us to examine bacterial replication and tissue damage following *Ft* SchuS4challenge. Although unvaccinated outbred mice showed extended MTD compared to unvaccinated inbred mice (Fig 1D), both groups exhibited similar bacterial burdens in the lungs and spleen (S2 Fig). Similarly, vaccinated outbred and inbred mice exhibited no difference in bacterial burden in the lungs and BALF until >7 days post-challenge. However, by day 10, a ~500-fold reduction in *Ft* SchuS4 was observed in outbred versus inbred mice (Fig 2A and 2B) at a time when the vaccinated inbred mice began to die (Fig 1C–1F). Remarkably, the spleens of vaccinated outbred mice displayed significantly lower bacterial loads throughout the infection regime, as compared to spleens of inbred mice (Fig 2C). Coincident with the rapid bacterial expansion on d 10, we observed severe lung inflammation associated with cellular infiltration and necrosis allied with protein flow into the BALF, signifying more severe tissue damage in the lungs of inbred mice (Fig 2D and 2E). Elevated serum LDH levels were also observed, suggesting more progressive disease and systemic inflammation in inbred mice (Fig 2F).

### Early and increased Th1/Th17-mediatedcytokine responses in outbred SW mice following *Ft* SchuS4 challenge

**Fig 3:**
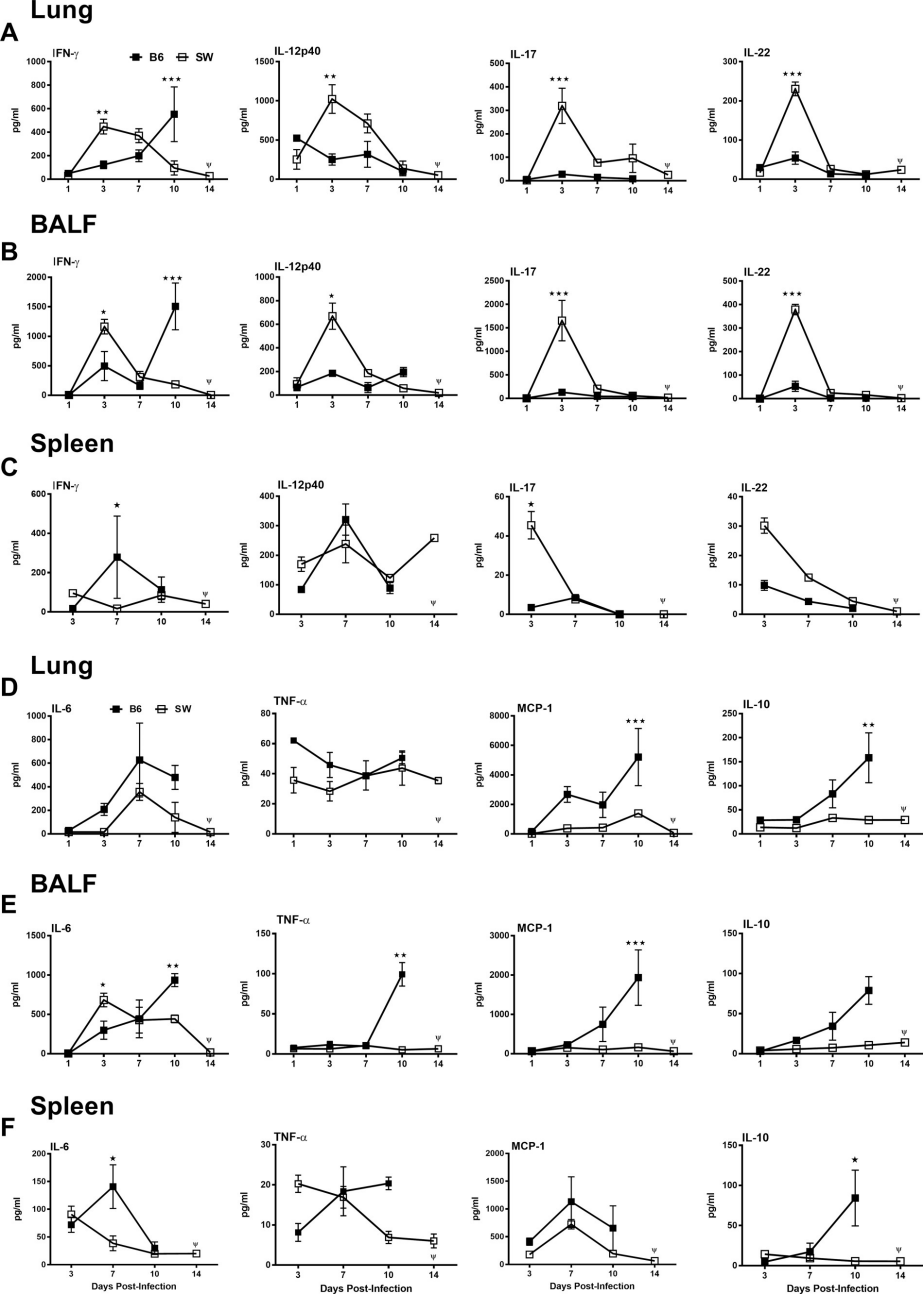
Early and strong Th1/Th17-mediated cytokine responses in outbred SW versus inbred C57BL/6mice were produced following *Ft* SchuS4 challenge. SW and C57BL/6 female mice were immunized and challenged as described in Fig 2. Lungs (**A & D**), BALF (**B & E**), and spleen (**C & F**) were then analyzed for the indicated cytokines on 1, 3, 7, 10, and 14 days post-challenge. Each point represents the average of 3 mice. Data shown are representative of two independent experiments. All vaccinated inbred mice died, as indicated by Ψ on the *x*-axis, *p < 0.05, **p < 0.01 and ***p < 0.001.

Our previous studies demonstrated that protected mice display augmented levels of Th1 and Th17 cytokines at early time points following *Ft* challenge [25, 26, 30]. In this study, outbred mice that showed superior protection against *Ft* SchuS4 also demonstrated elevated levels of IFN-γ and IL-12p40 during early infection in lungs, BALF and spleen. Vaccinated outbred mice also produced higher levels of IL-17 and IL-22 on day 3 after *Ft* SchuS4 challenge (Fig 3A–3C). In contrast, inbred C57BL/6 mice displayed increased pro-inflammatory cytokines coupled with IL-10, on day 10 post-challenge, after which the majority of inbred mice succumb to infection (Fig 3D–3F). Inbred mice also displayed increased IL-10, which likely inhibits the production of Th1 cytokines [31].

### Outbred SW mice exhibited rapid clearance of *Ft* LVS *sodB* mutant and a significantly lower Ab response

**Fig 4:**
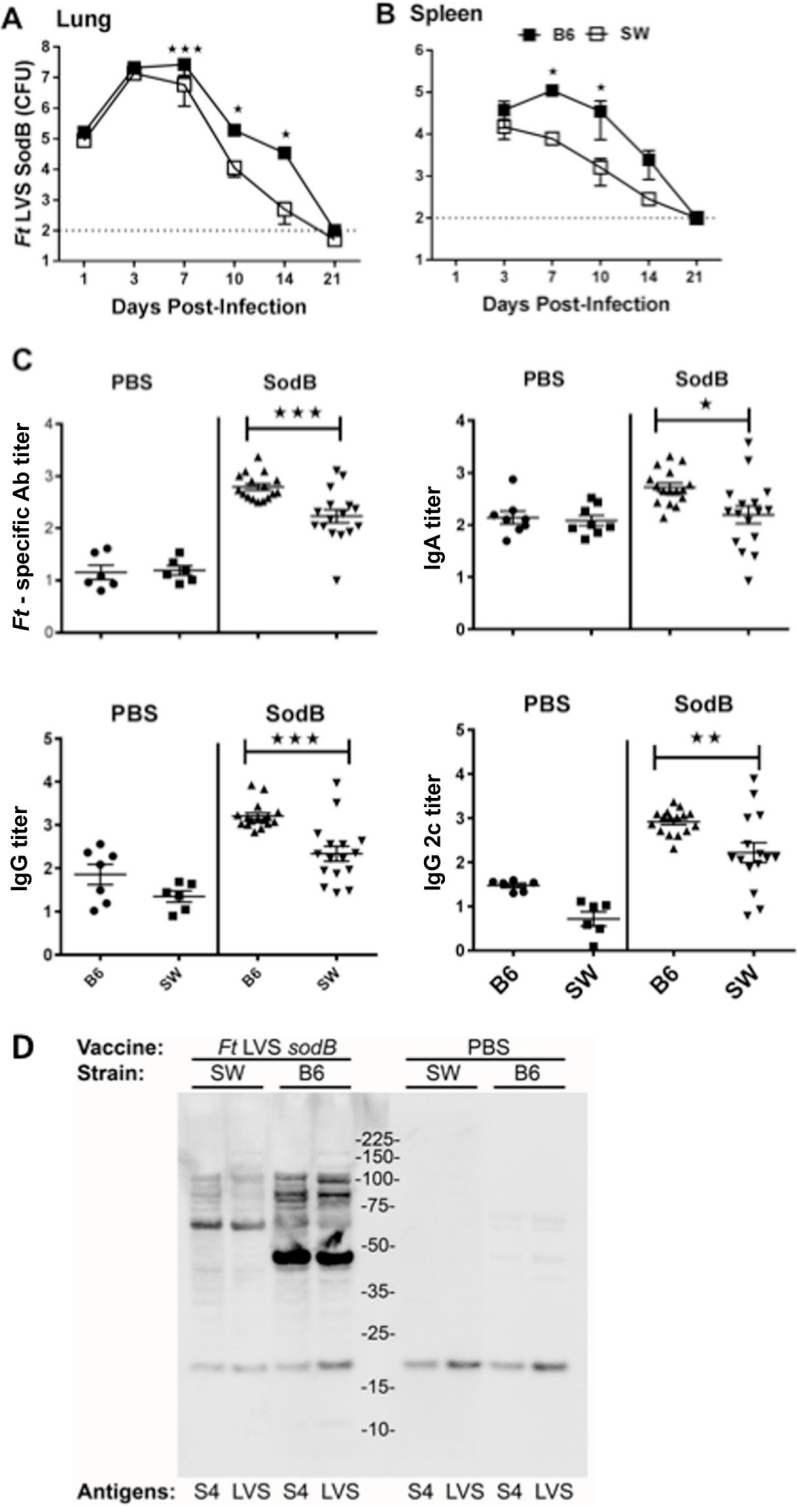
Outbred SW mice exhibited rapid clearance of *Ft sodB* mutant and significantly lower Ab response compared to that of inbred C57BL/6 mice. SW and C57BL/6 female mice were immunized as described in Fig 2. On days 1, 3, 7, 10, 14, and 21 bacterial loads (CFUs) in lungs (**A**) and spleen (**B**) were evaluated. Each point represents the average of 3 mice sacrificed per time point, *p < 0.05 and ***p < 0.001. Sera obtained 3 weeks post-immunization were analyzed for *Ft* specific total Ab, IgG, IgA, and IgG2c titers by ELISA. Data are represented as the mean ± SE (8 vaccinated and 4 unvaccinated mice per group) and are combined results from two independent experiments (**C**). Whole cell lysates of wild type *Ft* LVS and *clpB*-mutant SchuS4 grown in BHI broth were resolved by SDS-PAGE and transferred to a nitrocellulose membrane (**D**). Sections of the membrane, each containing one lane of LVS and one lane of SchuS4, were probed with one of the indicated 4 sera pools prior to chemiluminescent development. To account for the higher titer of specific Ab in the vaccinated, C57/BL6 mice (~500 vs ~250, Fig 4C), sera from SW mice were used in this case at a dilution of 1:1,000, whereas sera from the C57BL/6 mice were used at a dilution of 1:2,000. The ~18 kDa band visible in all lanes is an endogenously-biotinylated *Ft* protein (likely AccB) detected by the streptavidin-HRP conjugate used during development of western blots. Except for differences in the primary antibody, all the membranes shown were developed in parallel under identical conditions. *p < 0.05, **p < 0.01 and ***p < 0.001.

Next, we considered the role of bacterial vaccine growth kinetics in the responses we observed. Following vaccination with the *Ft* LVS *sodB* mutant, bacterial numbers recovered from the lungs and spleen of outbred mice were significantly lower than those observed in the tissues of similarly immunized inbred mice (Fig 4A and 4B).

Given that vaccinated outbred mice better survive mucosal *Ft* challenge we hypothesized that the Ab responses might also be distinct in outbred mice. Accordingly, we examined humoral immune responses in vaccinated mice. Remarkably, the Ab levels in outbred SW mice were 2–3 fold lower than that of inbred mice (Fig 4C), suggesting that, humoral immunity may not play a critical role in the increased protection observed in SW mice. However, further characterization of Ab responses by western blot analysis revealed marked qualitative differences in the serum Ab responses of inbred and outbred mice (Fig 4D). Specifically, vaccinated inbred mice induced a prominent Ig response towards a ~45 kDa *Ft* protein conserved in *Ft* LVS and *Ft* SchuS4 that is largely unrecognized by sera from vaccinated outbred mice. In addition, an ~ 60 kDa protein appears to be preferentially (though not exclusively) recognized by serum Ab from outbred mice. Additional *Ft* Ags in the ~85–120 kDa range are recognized in both mouse strains albeit to differing degrees. In contrast to Ab response, the splenic T cell response in the outbred mice following wildtype *Ft* vaccination was comparable with inbred counterpart (S3 Fig).

### CD4^+^ and CD8^+^ T cells and IFN-γ are essential, whereas IL-17A plays a minimal role during lethal pulmonary *Ft* SchuS4 infection in outbred SW mice

**Fig 5:**
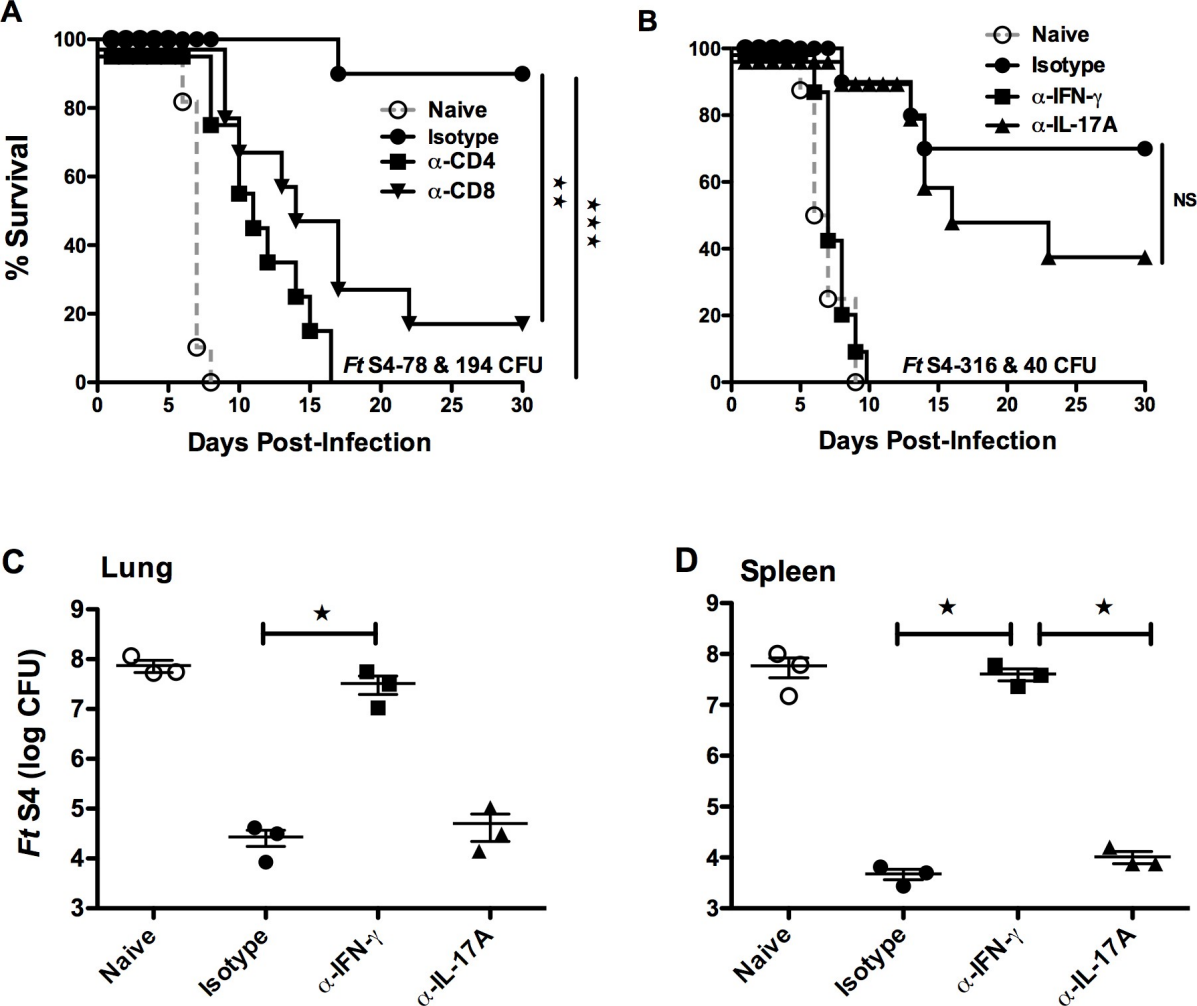
CD4^+^ and CD8^+^ T cells and IFN-γ are essential for surviving virulent *Ft* infection in outbred SW mice. SW female mice were immunized and challenged as described in Fig 2. Vaccinated mice were treated with anti-CD4^+^, anti-CD8^+^, or isotype control Ab on days -4, -1, 2, 5, and 8 relative to *Ft* SchuS4 infection and monitored for survival (**A**). Alternatively, mice were treated with anti-IFN-γ, anti-IL-17A, or isotype control Ab on days -1, 1, 3, and 5 relative to *Ft* SchuS4 infection and monitored for survival (**B**). *Ft* SchuS4 burdens in the lungs and spleens of naïve or vaccinated SW mice treated with depleting Ab specific for IFN-γ or IL-17A (n = 3 mice per group, day 5 post 40 CFU *Ft* SchuS4 in challenge) (**C** and **D**). Panels A & B represents combined results from two independent experiments with a total of 10 mice/ group, panel C is representative of two independent experiments. *p < 0.05, **p < 0.01, and ***p < 0.001.

Having observed a lower Ab response in vaccinated outbred mice, we next examined the cell-mediated immune response in *Ft* vaccinated outbred and inbred mice. Notably both inbred and outbred mice generated similar number of splenic CD4 and CD8 T cells in response to *Ft* vaccination (S3 Fig). However, T cell depletion in outbred mice following *Ft* SchuS4 challenge demonstrated that *sodB* vaccinated outbred mice depleted of CD4^+^ T cells were highly susceptible to *Ft* SchuS4 infection with a MTD of 11 days. CD8^+^ T cell-depleted mice succumb to infection with a slightly longer MTD (15 days) and a slightly higher survival rate (Fig 5A). The latter CD8^+^ T-cell-independent protective immunity was eliminated when the experiment was repeated with a higher challenge dose (78 versus 194 CFU, respectively).

Previous studies from our laboratory and others have also shown that vaccinated IFN-γ ^-/-^ and IFN-γ depleted inbred mice are highly susceptible to virulent *Ft* challenge, signifying IFN-γ importance in protective immunity to *Ft* infection [25, 32–34]. In contrast, the importance of IL-17A in protective immunity differs between non-virulent and virulent strains of *Ft* [35, 36]. Here we investigated if enhanced protection against *Ft* SchuS4 challenge in outbred SW mice could be abolished by IFN-γ or IL-17A neutralization. We found that *in vivo* blockade of INF-γ nullified the protection in vaccinated SW mice even with a substantially lower challenge dose (Fig 5B) and restored the bacterial load on day 5 to that naïve animals (Fig 5C). Precisely, we used two different (high and low) challenge doses to optimize our chances of detecting any differences due to cytokine depletion. In the case of IFN-γ depletion, regardless of challenge dose, the same result was observed in that all mice died mice against *Ft* emphasizing that IFN-γ is crucial for protective immunity in SW mice. In contrast, IL-17A neutralization had no significant impact on mortality or bacterial load, despite mice were challenged with higher infection dose (Fig 5B and 5C). Specifically, in the case of IL-17A depletion, the case between experiments was similar in that regardless of challenge dose, susceptibility of immunized SW mice to *Ft* S4 challenge was not significantly altered, even at an approximately 8-fold higher challenge dose (316 CFU).

## Discussion

Laboratory-adapted inbred mice have been used in the study of immunology and vaccine development for decades, but such responses are not always indicative of those of free-living, outbred animals such as humans. Comparison of the immune function of wild and laboratory mice has indicated that wild/outbred animals are more immunologically responsive, exhibit a more highly activated cellular immune system, yet also demonstrate a lower, more controlled cytokine response to pathogen-associated ligands compared to that of laboratory mice [37, 38]. Thus, in contrast to inbred rodent animal models in which breeding has been manipulated for the expression of a very limited MHC repertoire, it is reasonable to predict that use of outbred species may be more predictive in the identification of protective vaccine antigens and immune responses for an outbred human population. Importantly, in this case, we chose an outbred mouse strain to model the immune response and protection following vaccination in an outbred population. We chose this model because we believe it is more likely to reflect the dynamic immune interactions and responses that would occur in the presence of multiple MHC haplotypes present in an outbred population. However, other published work has also indicated that such studies may be done using multi-strain, inbred mice, which can achieve statistical significance with fewer animals [39]. However, we initiated this study to further develop the outbred SW vaccine model and examine the level of protection in vaccinated outbred SW mice versus inbred C57BL/6 mice.

We observed that in the case of primary i.n. *Ft* infection, outbred SW mice are slightly more resistant to tularemia than inbred C57BL/6 mice (Fig 1A–1C). In contrast, vaccinated outbred and inbred mice demonstrated a significant differential protection against both *Ft* LVS and *Ft* SchuS4 challenge. Specifically, in agreement with earlier observations [23, 25, 26], mucosal vaccination using i*Ft* or *Ft* LVS *sodB* in inbred mice induced moderate protection against *Ft* LVS or low dose *Ft* SchuS4 challenge, respectively (Fig 1A–1C), whereas no protection was observed against high challenge dose infections (Fig 1D–1F). In contrast, i*Ft* or live *sodB* vaccinated outbred mice showed complete protection against *Ft* LVS and SchuS4 challenge, respectively. Furthermore, a single dose *sodB* immunization induced long lasting immunity against the highly virulent *Ft* SchuS4 strain 90 days post vaccination. This contrasts to earlier studies in which intradermal *Ft* vaccination failed to protect outbred SW mice against virulent *Ft* strain #33 systemic challenge [21]. There may be a number of potential explanations for this dichotomy, they include: differences in vaccine administration, the vaccination regime, variations in the immune response to wildtype versus *sodB* mutant vaccine, or differential immunological requirements for protection against intradermal versus intranasal *Ft* SchuS4 challenge. Extensive additional research will be required to identify the exact cause for this difference.

The reduced susceptibility of vaccinated outbred mice to *Ft* SchuS4 infection correlates with reduced weight loss, lower pathogen burden, and less tissue inflammation following *Ft* SchuS4 challenge. Specifically, C57BL/6 inbred mice displayed, increased weight loss, robust expansion of *Ft*, and increased tissue inflammation in the lungs versus SW mice (Fig 2A–2D). *Ft* SchuS4 expansion in the lungs of inbred mice at day 10 correlated with heightened cytokine responses and severe tissue damage suggesting that uncontrolled bacterial growth coincides with a lethal acute inflammatory response [40]. The severe tissue inflammation was also reflected in increased serum LDH and BALF total protein levels (Fig 2E and 2F). This is consistent with the idea that pulmonary infections causes more severe tissue damage in C57BL/6 inbred mice making them more susceptible to a lethal infection [41]. We further observed that outbred mice displayed effective clearance of *Ft* SchuS4 in the spleens from the early time point demonstrating strong protective immunity generated in peripheral organs in vaccinated outbred mice, as has also been observed in BALB/C mice [42]. Taken together, the earlier/more potent splenic immune response (IFN-γ and TNF) in SW mice may also explain why SW mice are better protected.

We have previously demonstrated that mice protected against *Ft* via vaccination display increased Th1 (IL-12 and IFN-γ) cytokines early in infection [25, 26, 30]. Similar cytokine patterns were also observed in vaccinated outbred SW mice, which were coupled with a robust production of IL-17A and IL-22 cytokines (Fig 3A–3C). Outbred mice also displayed significantly less IL-6, MCP-1, and TNF-α expression throughout infection. Interestingly, the aforementioned cytokines, which are indicators of systemic inflammation, illness, and sepsis [43], were elevated substantially in inbred mice (Fig 3D–3F). Inbred mice also displayed increased IL-10, which tends to inhibit production of IL-12 and other Th1 cytokines including IFN-γ and IL-2 [31]. This implies that C57BL/6 mice favor the development of a Th2 phenotype in the lung versus the more protective Th1 response [4, 9]. The analysis of Th2 cytokines and their relationship to survival (beyond correlation) is currently an active area of investigation in our laboratory.

The role of humoral immunity in protection against *Ft* remains controversial. Specifically, humoral immunity alone has been shown to be protective against *Ft* LVS infection, but not against virulent *Ft* S4 infection, which requires both humoral and cellular immune responses [23, 25, 44, 45]. In this study, well-protected outbred SW mice exhibited lower Ab levels compared to their inbred counterparts and this Ab also differed qualitatively to that of inbred C57BL/6 mice (Fig 4C and 4D). Thus, although quantitative differences in Ab levels between outbred and inbred mice suggest Ab is not central to protection of the outbred SW mice, qualitative differences could still play an important role.

We further hypothesized that the increased protection against *Ft* SchuS4 in outbred SW mice could be T cell-mediated, as has been demonstrated in inbred mouse models [23, 27, 33]. When we examined the cell-mediated immune response following *Ft* vaccination both inbred and outbred mice generated similar number of splenic CD4 and CD8 T cells (S3 Fig). Nevertheless, depletion of CD4^+^ and CD8^+^ T cells abolished protection against *Ft* SchuS4 challenge in vaccinated outbred SW mice (Fig 5A). CD4+ T cell-depleted mice were more susceptible to *Ft* SchuS4 challenge than CD8+ T cell-depleted mice, implying CD4+ T cells play the crucial role in vaccine-induced protection in SW mice as demonstrated for C57BL/6mice [27].

The significance of IFN-γ in protective immunity to *Ft* infection is well established in inbred BALB/c and C57BL/6 mice [25, 33, 34]. Consistent with this, IFN-γ depletion in vaccinated outbred mice exhibited heightened sensitivity to *Ft* S4 infection (Fig 5B and 5C) signifying an important role in protection against *Ft* SchuS4 infection in SW mice as well. In contrast, IL-17A neutralization did not alter the susceptibility of immunized outbred mice to *Ft* SchuS4 challenge, which is in general agreement with earlier mice studies, although the significance of IL-17A in protective immunity differs between LVS and SchuS4 [34–36, 46].

**Fig 6:**
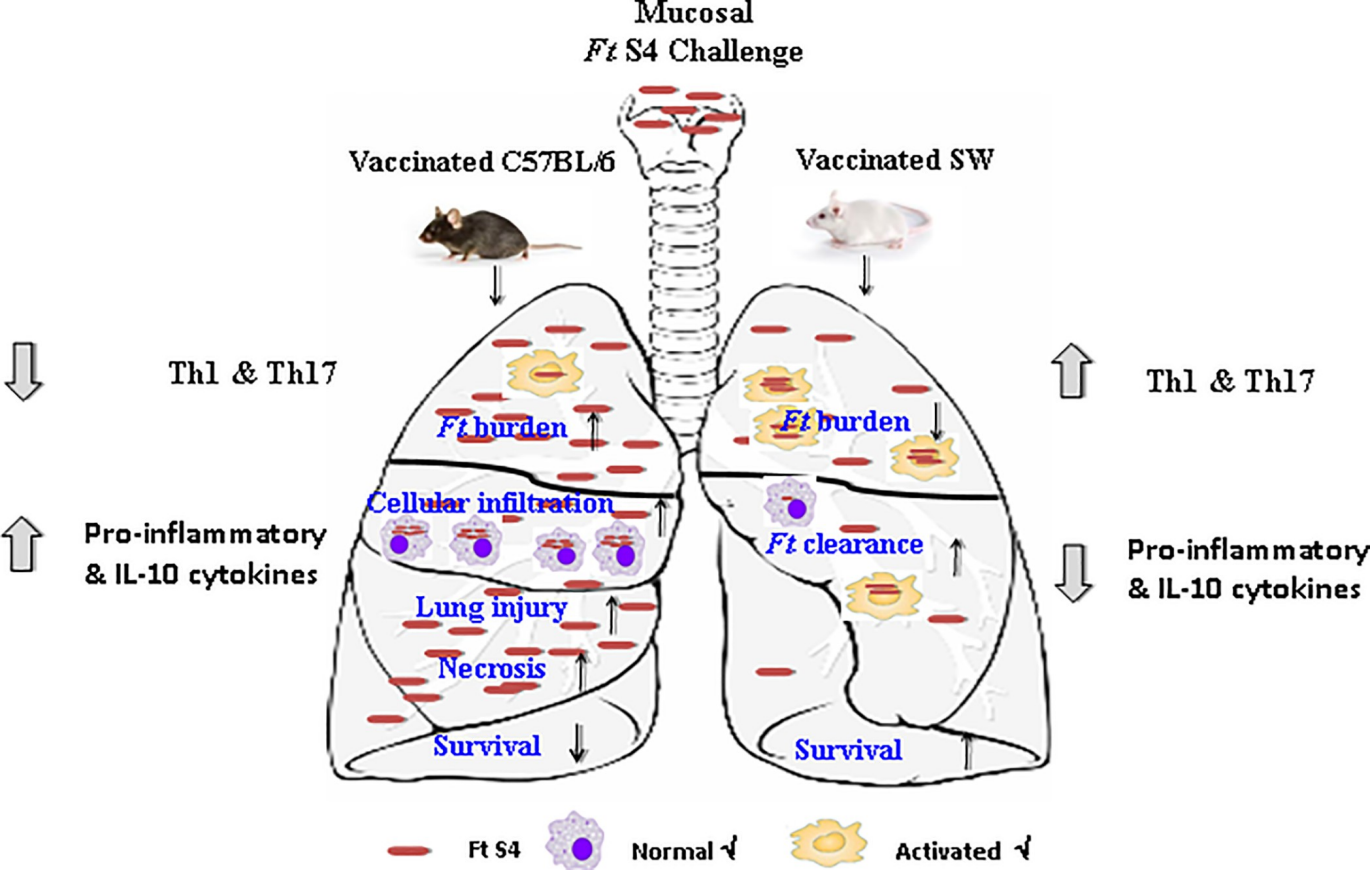
A schematic representation of tularemia pathogenesis in vaccinated inbred C57Bl/6 versus outbred SW Mice. Vaccinated outbred SW and inbred C57BL/6 mice handle the progression and outcome of tularemia disease in different ways. Vaccinated SW mice in comparison to C57BL/6 mice engage early Th1 and Th17 cytokines, along with controlled production of pro-inflammatory cytokines with reduced inflammation. Whereas vaccinated C57BL/6 mice displayed robust expansion of *Ft* Schus4 with severe lung inflammation associated with cellular infiltration and necrosis allied with protein flow into the BALF, signifying more severe tissue damage in the lungs.

In summary, we demonstrate the use of an outbred mouse model in *Ft* vaccine studies. Our results also suggest that both outbred SW and inbred C57BL/6 mice handle the progression and outcome of tularemia disease in different ways, suggesting that host genotypes play a major role in the outcome of tularemia infection (Fig 6). Interestingly, the protective immune responses in outbred SW mice were similar to that of inbred BALB/c mice suggesting both mouse strains could serve as models for *Ft* vaccine development, the latter being particularly valuable when requiring knockout mice. As discussed, an alternative approach to broadening the genetic diversity within the mouse model for vaccine development also could include designed multi-strain, inbred studies, which can achieve statistical significance with fewer animals than outbred studies [39]. However, these studies also suggest, given the lethality of *Ft* SchuS4 to inbred mouse strains and the MHC diversity of SW mice, that the ability to vaccinate and challenge outbred SW mice with higher *Ft* SchuS4 doses in the context of a more diverse MHC repertoire may provide significant advantages in terms of dosing and efficacy studies to that of Balb/c mice. The use of an outbred mouse model to study *Ft* vaccine efficacy is likely more predictive of protective potential for an outbred human population.

## Supporting information

S1 Fig Outbred SW male and female mice are equally protected against respiratory *Ft* SchuS4 challenge. SW and C57BL/6 male and female mice were immunized i.n. with 1x10^3^ CFU of attenuated *Ft* LVS *sodB* mutant mice were then challenged i.n. with 146 CFU of *Ft* SchuS4 on day 21 and monitored for survival for 30 days.
(TIF)

S2 Fig Bacterial burdens in the lungs of vaccinated, outbred mice following SchuS4 challenge are lower than those of inbred mice. SW and C57BL/6 female mice were immunized i.n. with 1x10^3^ CFU of attenuated *Ft* LVS *sodB* mutant. Mice were then challenged on day 21 i.n. with 42 CFU of *Ft* SchuS4. Lungs (**A**) and spleen (**B**) were analyzed for bacterial burdens on 5 days post-challenge. Each point represents the mean +/- SE of 3 mice sacrificed per time point, these data are representative of two independent experiment of total of six mice.
(TIF)

S3 Fig The splenic T cell response in the outbred mice following wildtype *Ft* vaccination was comparable with inbred counterpart. SW and C57BL/6 female mice were immunized i.n. with 1x10^3^ CFU of attenuated *Ft* LVS *sodB* mutant. Single-cell suspensions of splenocytes from SW and C57BL/6 female mice (*n* = 3) were generated. Cells were counted and then stained for surface expression of CD4, CD8 and analyzed by FACS. Total splenocyte counts **(A)** and absolute cell counts of CD4+ and CD8+ cells percentage were determined **(B)**. Data are representative of two independent experiments.
(TIF)

## Abbreviations

Ft: *F*. *tularensis*
i*Ft*: inactivated *Ft*
sodB: superoxide dismutase B
Ab: antibody
i.n.: intranasal
i.d.: intradermal
BALF: bronchial alveolar lavage fluid
MTD: median time to death
SW: Swiss Webster
S4: SchuS4
LVS: live vaccine strain
MHC: major histocompatibility complex
LDH: lactate dehydrogenase
