# Supplementary figures and images for: Evaluation of an outbred mouse model for Francisella tularensis vaccine development and testing
Source: PLoS One. 2018 Dec 11;13(12):e0207587. doi: 10.1371/journal.pone.0207587 (PMC6289435; doi:10.1371/journal.pone.0207587)

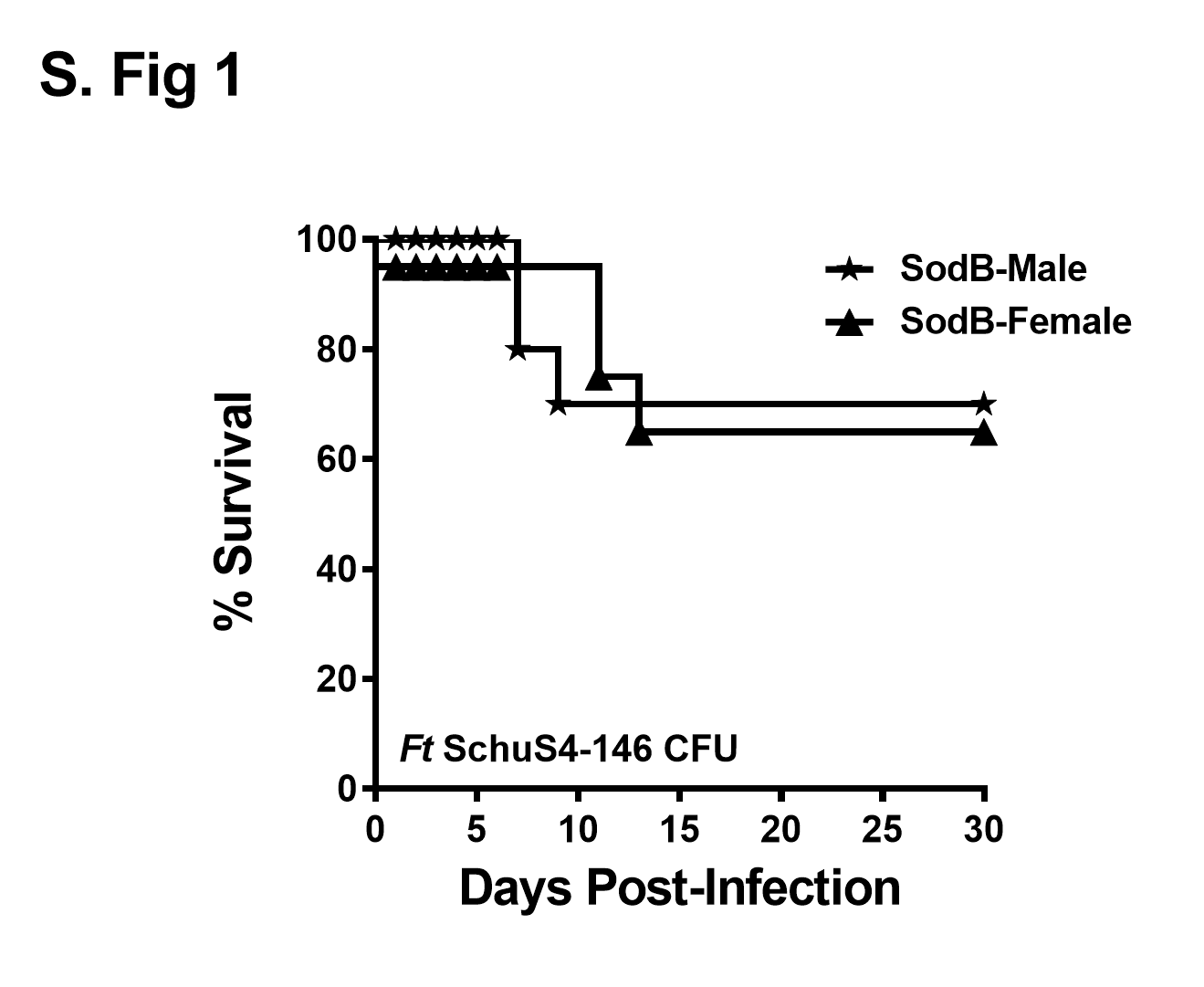

Supplement: S1 Fig — SW and C57BL/6 male and female mice were immunized i.n. with 1x10^3^ CFU of attenuated Ft LVS sodB mutant mice were then challenged i.n. with 146 CFU of Ft SchuS4 on day 21 and monitored for survival for 30 days. (TIF) [file pone.0207587.s001.tif]

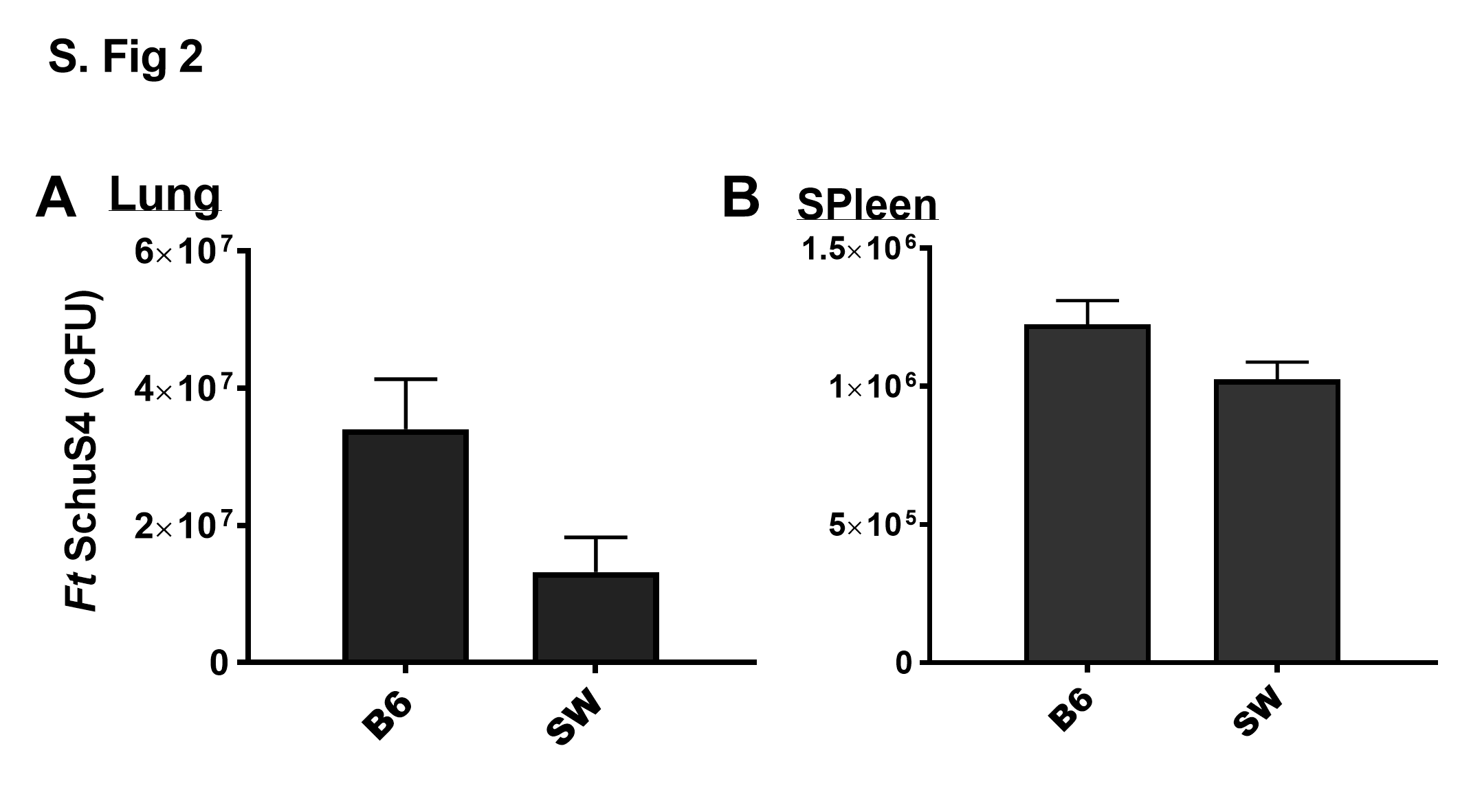

Supplement: S2 Fig — SW and C57BL/6 female mice were immunized i.n. with 1x10^3^ CFU of attenuated Ft LVS sodB mutant. Mice were then challenged on day 21 i.n. with 42 CFU of Ft SchuS4. Lungs (A) and spleen (B) were analyzed for bacterial burdens on 5 days post-challenge. Each point represents the mean +/- SE of 3 mice sacrificed per time point, these data are representative of two independent experiment of total of six mice. (TIF) [file pone.0207587.s002.tif]

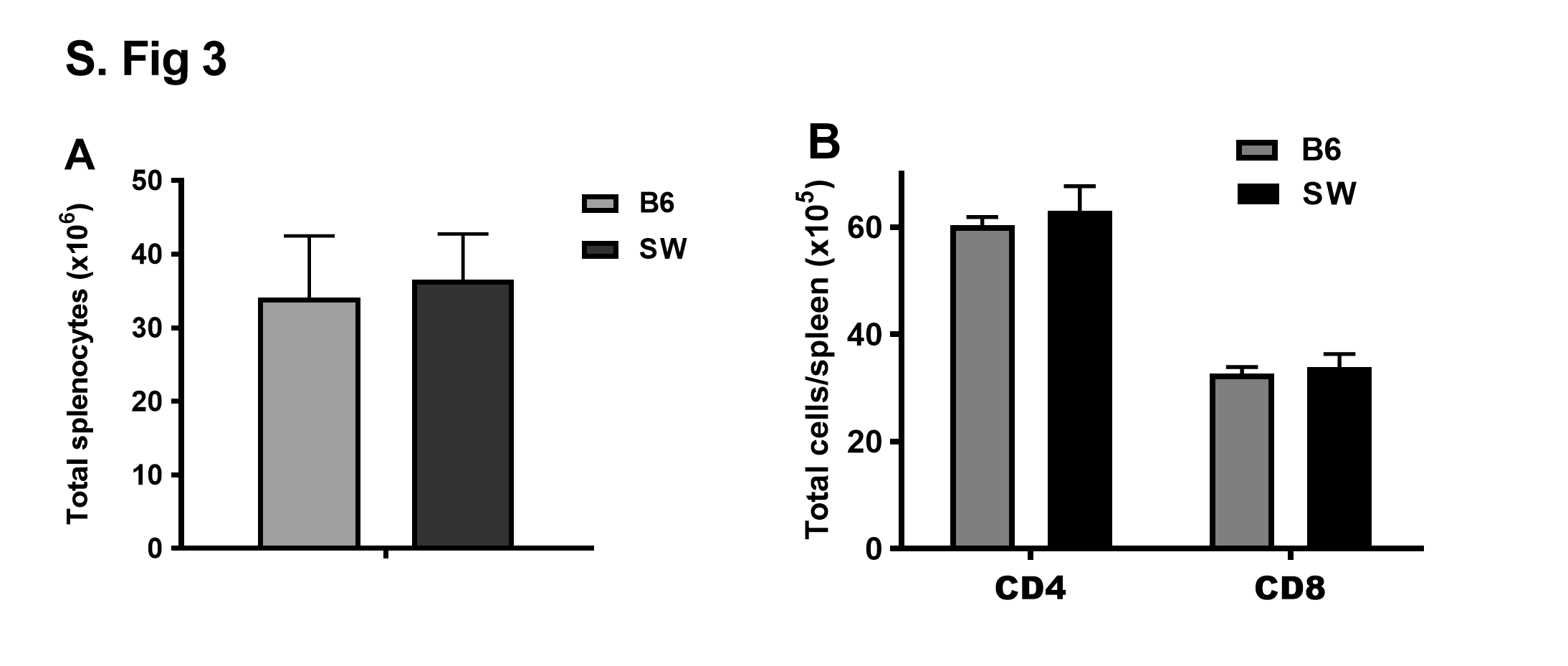

Supplement: S3 Fig — SW and C57BL/6 female mice were immunized i.n. with 1x10^3^ CFU of attenuated Ft LVS sodB mutant. Single-cell suspensions of splenocytes from SW and C57BL/6 female mice (n = 3) were generated. Cells were counted and then stained for surface expression of CD4, CD8 and analyzed by FACS. Total splenocyte counts (A) and absolute cell counts of CD4+ and CD8+ cells percentage were determined (B). Data are representative of two independent experiments. (TIF) [file pone.0207587.s003.tif]
